# Supplementary material for: Multitarget Reactive Magnetron Sputtering towards the Production of Strontium Molybdate Thin Films
Source: Materials (Basel). 2023 Mar 8;16(6):2175. doi: 10.3390/ma16062175 (PMC10056819; doi:10.3390/ma16062175)
Supplement: Supplementary file 1 [file materials-16-02175-s001.zip › materials-2141224-supplementary.pdf]

Supplemental Material

Multitarget Reactive Magnetron Sputtering Towards the Production of Strontium Molybdate Thin Films

Mindaugas Andrulevičius [1], Evgenii Artiukh [2], Gunnar Suchaneck [2], Sitao Wang [2], Nikolai A. Sobolev [3,*], Gerald Gerlach [2], Asta Tamulevičienė [1], Brigita Abakevičienė [1] and Sigitas Tamulevičius [1]

[1] Institute of Materials Science, Kaunas University of Technology, K. Baršausko St. 59, LT-51423 Kaunas, Lithuania
[2] Solid State Electronics Laboratory, TU Dresden, 01062 Dresden, Germany
[3] Departamento de Física and i3N, Universidade de Aveiro, 3810-193 Aveiro, Portugal
* Correspondence: sobolev@ua.pt

The survey spectra of all samples were found to be similar. The spectrum of sample #260719 (14.8% Mo) in Figure S1 shows a more pronounced Sr 3d peak due to a higher strontium concentration.

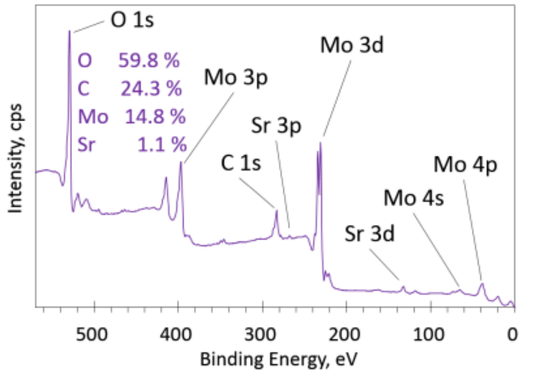

Figure S1. Survey XPS spectrum of the film containing 14.8 at.% Mo.

A comparison of normalized-intensity high-resolution Mo 3d XPS spectra before deconvolution for all Mo concentrations is presented in Figure S2. Differences in the Mo 3d spectra in this picture indicate the presence of lower-valence states of molybdenum for some samples.

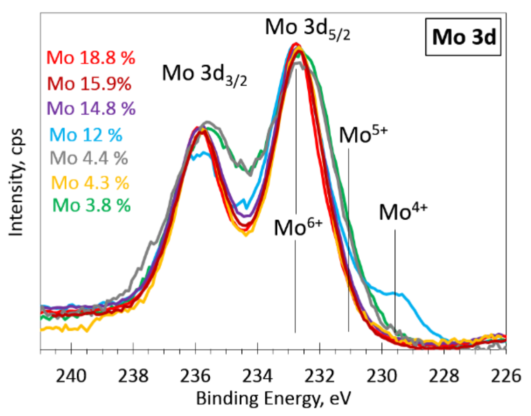

Figure S2. Comparison of normalized-intensity high-resolution Mo 3d XPS spectra for samples with various Mo concentrations.

A comparison of high-resolution Mo 3d XPS spectra for all samples with that of sample #310719 (Mo 12.0%) is presented in Figure S3. The Mo 3d spectrum of sample #310719 was used as a reference since it clearly exhibited the presence of lower-valence peaks of molybdenum oxide. For all samples (except sample #310719), only the Mo $3d_{5/2}$ peak fitting is shown in this picture for clarity.

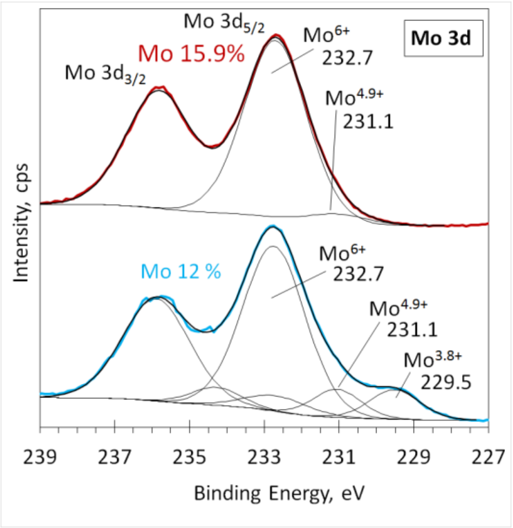

a)

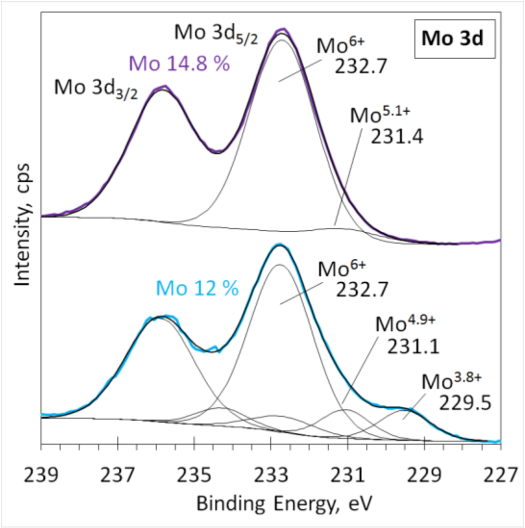

b)

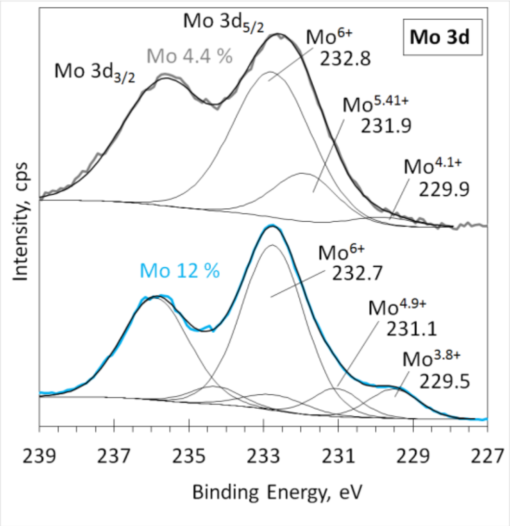
Mo 3d$_{5/2}$
Mo 3d$_{3/2}$
Mo 4.4 %
Mo^{6+}
232.8
$Mo^{5.41+}$
231.9
$Mo^{4.1+}$
229.9
Mo 3d
Mo 12 %
Mo^{6+}
232.7
$Mo^{4.9+}$
231.1
$Mo^{3.8+}$
229.5
Intensity, cps
239
237
235
233
231
229
227
Binding Energy, eV

c)

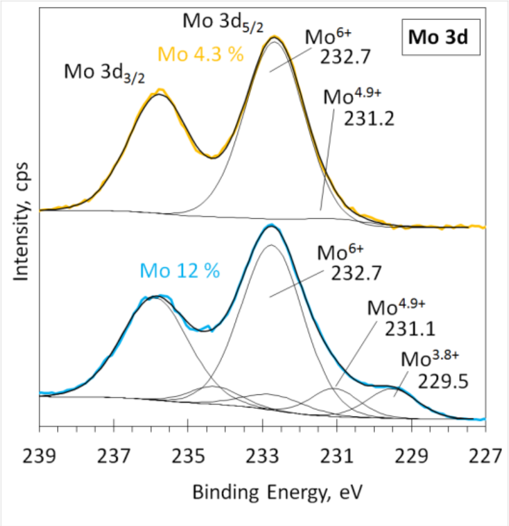
Mo 3d$_{5/2}$
Mo 3d$_{3/2}$
Mo 4.3 %
Mo^{6+}
232.7
$Mo^{4.9+}$
231.2
Mo 3d
Mo 12 %
Mo^{6+}
232.7
$Mo^{4.9+}$
231.1
$Mo^{3.8+}$
229.5
Intensity, cps
239
237
235
233
231
229
227
Binding Energy, eV

d)

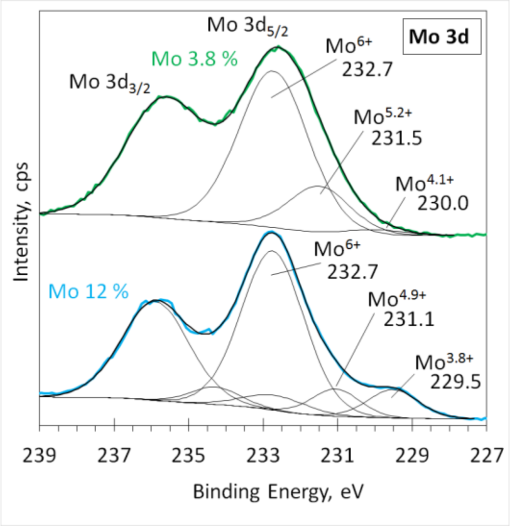
Mo 3d$_{5/2}$
Mo 3d$_{3/2}$
Mo 3.8 %
Mo^{6+}
232.7
$Mo^{5.2+}$
231.5
$Mo^{4.1+}$
230.0
Mo 3d
Mo 12 %
Mo^{6+}
232.7
$Mo^{4.9+}$
231.1
$Mo^{3.8+}$
229.5
Intensity, cps
239
237
235
233
231
229
227
Binding Energy, eV

e)

Figure S3. Comparison of high-resolution Mo 3d XPS spectra for all samples with those of sample #310719 (12.0% Mo).

Normalized Sr 3d spectra for samples with different Mo concentrations are presented in Figure S4. From this picture, one can see that the Sr 3d spectra for these samples are very similar. The deconvolution of all spectra resulted in two overlapping peaks of a typical Sr 3d spin-orbit doublet. The peak fitting results are shown only for the sample containing 3.8% Mo (Figure S4, spectrum at the bottom), not to overload the figure.

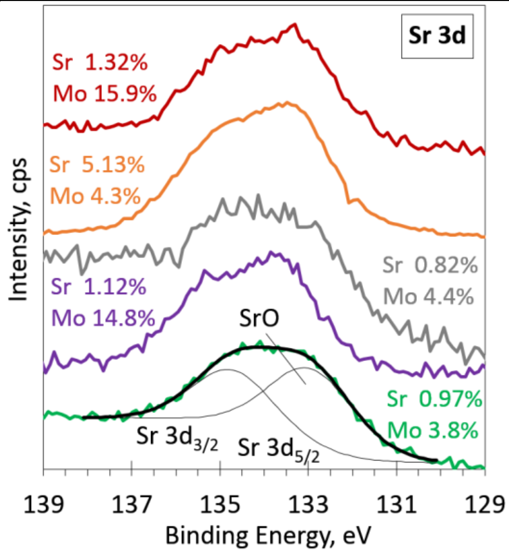

Figure S4. Normalized-intensity high-resolution Sr 3d XPS spectra for samples with different Mo concentrations.

Comparison of the oxygen XPS spectra in the O 1s region (Figure S5) showed negligible differences between samples. Deconvolution of all spectra resulted in two peaks – a high-intensity peak at approximately 530.5 eV and a lower-intensity peak at approximately 532.3 eV. We attributed the main peak at 530.5 eV to oxygen-molybdenum bonds – its position corresponds to the known peak position for different molybdenum oxides: MoO_3 (530.65 eV), MoO (530.5 eV), and MoO_2 (530.45 eV) [11]. It should be considered that the known binding energy values for the O-C=C bonds are very similar (531.0 – 531.4 eV) [11], and the peaks for these bonds could overlap with the main peak. The peak of lower intensity at ca. 532.3 eV may be attributed to the C-O-C bonds (532.8 eV) with a possible overlap of the O-H bonds (533 eV) [11,12] from atmospheric contaminants. The peak fitting is shown only for the sample containing 14.8% Mo for clarity (Figure S5, spectrum at the bottom).

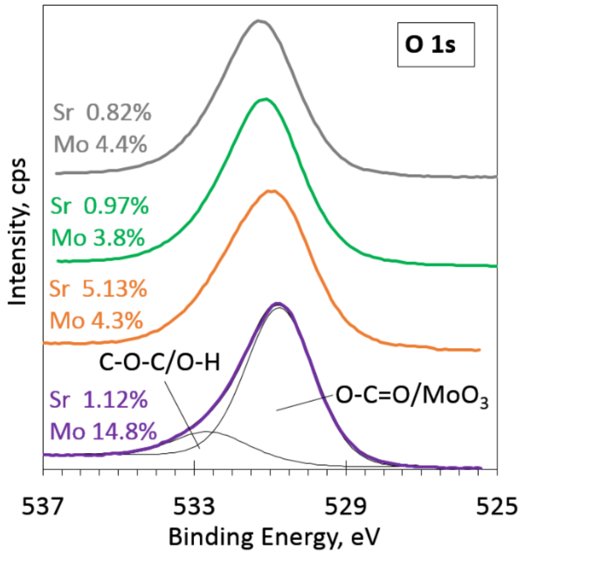

Figure S5. Normalized-intensity high-resolution O 1s XPS spectra for samples with different Mo concentrations.

Table S1. Relative concentration of Mo bonds for all samples calculated by means of spectra deconvolution.

Mo bond	#140819	#150819	#260719	#310719	#020819	#120819	#250719
Mo^{4+}, %	0	0	0	11.2	2.4	4.0	0
Mo^{5+}, %	10.1	5.7	11	10.2	20.3	20.3	5.3
Mo^{6+}, %	89.9	94.35	89.0	78.6	77.2	75.7	94.7

The XRD pattern of different samples and the Al_2O_3 substrate are shown in Figure S6. In the XRD patterns of samples #260719, #310719, #020819 and #120819 no visible peaks corresponding to molybdenum oxide were detected, and only substrate-related peaks of Al_2O_3 were identified.

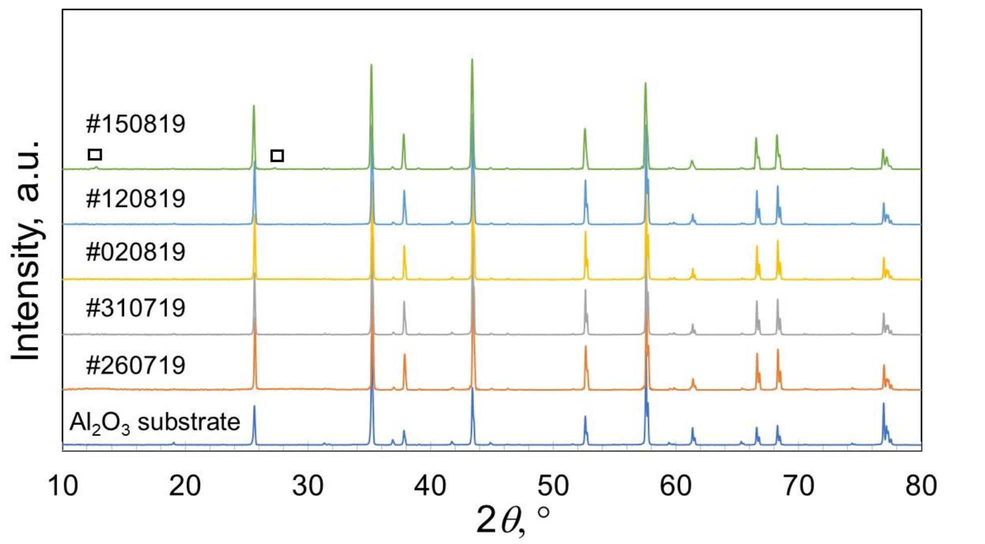

Figure S6. XRD patterns of samples #150819, #120819, #020819, #310719, #260719, and Al_2O_3 substrate. The peaks corresponding to the MoO_3 phase are indicated by squares.
